# Concussion Assessment and Management Self-efficacy Among Irish Clinicians

**DOI:** 10.1177/19417381241287209

**Published:** 2024-10-24

**Authors:** Anna P. Postawa, Siobhán O’Connor, Enda F. Whyte

**Affiliations:** † Centre for Injury Prevention and Performance, School of Health and Human Performance, Dublin City University, Dublin, Ireland; ‡ SHE Research Centre, Department of Sport and Health Science, Technological University of the Shannon - Midlands, Athlone, Ireland

**Keywords:** clinical practice, concussion care, self-efficacy, sport-related concussion

## Abstract

**Background:**

This study explored concussion assessment and management self-efficacy and practices of allied healthcare professionals in Ireland.

**Hypotheses:**

(1) Self-efficacy levels and practices vary across different concussion assessment and management skills, (2) the ability to practice skills impacts self-efficacy most.

**Study Design:**

Cross-sectional.

**Level of Evidence:**

Level 3.

**Methods:**

Survey of allied healthcare professionals (285 responders), investigating (1) demographics, (2) concussion assessment (immediate and office) and management (postconcussion advice and management/rehabilitation) self-efficacy levels and practices, and (3) factors affecting self-efficacy.

**Results:**

Levels of self-efficacy among clinicians were 64.5 ± 26.6 (immediate assessment) and 56.6 ± 25.4 (postconcussion advice) (highest scores: concussion symptom checklist [80 ± 28.4], physical rest advice [80.1 ± 27.8]; lowest: Child Sport Concussion Assessment Tool [44.6 ± 41.2] and nutrition advice [34.1 ± 33.7]). Overall levels of self-efficacy among Certified Athletic Therapists and Chartered Physiotherapists were 51.5 ± 20.1 (assessment) and 62.1 ± 20.9 (management) (highest scores: history/clinical evaluation nonspecific to concussion [86.6 ± 16.2], physical rest advice [86.3 ± 20]; lowest: paper/pencil neuropsychological test [16.7 ± 28.6], advice on medication use [39.2 ± 35]). A strong positive correlation was observed between clinician self-efficacy and frequency of use of overall (*r* = 0.795; *P* < 0.01) and immediate (*r* = 0.728; *P* < 0.01) assessment, advice (*r* = 0.805; *P* < 0.01), and management (*r* = 0.812; *P* < 0.01) skills. Factors with greatest positive impact on clinician self-efficacy were the ability to practice skills during clinical placement (3.3 ± 0.9) and remaining emotionally (3.3 ± 0.8) and physically (3.3 ± 0.8) calm while practicing.

**Conclusion:**

Clinicians in Ireland had moderate self-efficacy in concussion care. Those who used concussion-relevant skills frequently in practice displayed higher self-efficacy for those skills.

**Clinical Relevance:**

Concussion-related self-efficacy can be enhanced through practice in a clinical environment and through experiencing composure while practicing.

Concussion is a global public health concern and a major issue in sport.^46^ Timely recognition and appropriate management may mitigate its negative outcomes.^8^ Sport-related concussion (SRC) is considered one of the most complex injuries in sport, with its diagnosis, assessment, and management posing a challenge to healthcare practitioners.^42^

Best practice recommends adherence to the latest SRC assessment and management recommendations, eg, comprehensive baseline testing, multimodal concussion assessment, and staged return-to-play (RTP).^47^ However, lack of full compliance with these guidelines has been established in multiple countries and among various clinician groups.^36,37,41,72^ Absence or limited baseline testing^47^; limited use of 3-domain minimum concussion assessment and reassessment^12,36,37,41^; and poor implementation rate of vestibular, balance, and graded exertional training in concussion management were the main issues reported.^72^ In Ireland, limited use of 3-domain minimum concussion assessment/reassessment was also identified among athletic therapists,^36^ with general lack of awareness of current concussion-related recommendations and poor practices established among chartered physiotherapists.^43^ So far, shortage of funding,^36^ time, and delay in dissemination of the latest consensus statement recommendations by the local sporting organizations and governing bodies^43^ have been reported as the main reasons for poor compliance with gold standard practice in Ireland. However, a qualitative investigation of barriers to its implementation is worth exploring. Limited curriculum, lack of staff and their inadequate training,^37^ shortage of equipment, and cultural barriers were reported in other countries.^57^ Gaps in concussion-relevant theoretical and practical education are well documented in literature across different healthcare professional and student groups,^25,30,35,40,58,68^ with a poor level of concussion-related knowledge evident worldwide.^9,25,32,35,65^ Knowledge acquired through education is not the only factor that impacts healthcare professionals’ practice.^72^ Attitudes and personality traits have been shown to strongly influence behavior. However, human actions in specific contexts are impacted through them only indirectly.^1^ Context-specific behavior, as explained by the theory of planned behavior, is impacted primarily by the intention to demonstrate the behavior, which in turn is influenced by perceived behavioral control, also known as self-efficacy.^1,72^ Self-efficacy, established as a part of social cognitive theory, is defined as a personal belief in one’s own capability to perform a specific task successfully. Its level is suggested to influence a person’s choices, persistence, and performance in the skill it relates to,^63^ by either motivating to engage or inclining to avoid and give up quickly in the face of difficulty.^62^ Thus, the level of self-efficacy regarding concussion care may impact healthcare professionals’ practices.^61,72^ The overall level of American athletic trainers’ (ATs) self-efficacy in assessment and management of concussion is moderate, with ratings for specific assessment/management techniques ranging from very low to high. This might explain, at least partially, the reason for their noncompliance with concussion gold standard recommendations.^61^ Investigation of American physical therapists’ self-efficacy indicated high and moderate levels of general confidence in concussion recognition, management, and ability to provide clearance for return to sport.^72^ Critically, no specific concussion assessment or management skills were included in the study, so the findings only reflect on clinicians’ overall confidence beliefs, and not self-efficacy, which is task specific,^48^ and is a strong predictor of human behavior.^49^

The general sources of self-efficacy are well known and include mastery experience, vicarious experience, verbal persuasion, and physical and emotional arousal.^5^ However, research on the factors that impact development of self-efficacy in practical skills among healthcare professionals is scarce.^67^ The existing evidence suggests that mastery experience and observation of role models impact medical students’ self-efficacy.^18,66,72^ Role models were suggested to stimulate self-efficacy not only through demonstration of skill, but also through provision of feedback and encouragement to further practice.^66^ The quality of practical experience appears to be crucial, with practice in a clinical environment shown to impact undergraduate nursing students’ self-efficacy in family nursing skills,^29^ and high-fidelity simulation improving athletic training students’ self-efficacy in emergency cardiovascular care skills.^50^ Considering the importance of self-efficacy for clinical practice, it seems crucial to establish how to facilitate its development during professional education.

To date, no research has examined the sources of concussion assessment/management self-efficacy, its relationship with clinical and field-based patient care, and no comparison exists for self-efficacy or practices across healthcare professional groups. In addition, concussion assessment and management self-efficacy has never been explored in an Irish context. Thus, this study aimed to (1) identify the levels of self-efficacy in concussion assessment and management, (2) explore the relationship between self-efficacy and clinical/field-based practices, and (3) investigate the factors that impact the development of self-efficacy among clinicians most commonly involved in a team sports’ athlete care in Ireland (certified athletic therapists [CATs], chartered physiotherapists [ChPs], emergency medical services practitioners [EMSPs]).

## Methods

### Participants

A cross-sectional study design was implemented. Eligible participants were allied healthcare professionals (CATs, ChPs, and EMSPs), currently working in Ireland. Participants were excluded if not currently practicing in Ireland or if they did not assess or manage concussion as part of their clinical practice. Raosoft sample-size calculator indicated a minimum sample size of 267 (5% margin of error, 90% CI).^22^ Only participants who completed ≥1 section of the survey apart from the demographics were included in data analysis.

### Instrumentation

An anonymous online survey (Appendix 1, available in the online version of this article), adapted from a previously published survey, investigating ATs’ self-efficacy in concussion assessment and management in the United States, was utilised.^61^ Questions were adapted for the Irish context and to reflect the latest concussion assessment and management recommendations.^13,19,20,31,34,39,70^ The survey consisted of 71 to 79 questions depending on whether a participant held postgraduate qualifications, was working with sporting populations, and had completed concussion-focused continuing professional development courses in the past. It consisted of 4 sections including participant demographics (Section 1), factors positively affecting self-efficacy (Section 2), factors negatively affecting self-efficacy (Section 3), and concussion assessment and management skills’ self-efficacy (Section 4). Sections 2 and 3 were added to investigate the impact that self-efficacy influencing factors, suggested by social cognitive theory,^4^ have on development of self-efficacy in concussion assessment and management.

Section 1 of the survey included questions on participant’s age, gender, past undergraduate and postgraduate education, inclusion of concussion assessment and/or management in professional education curriculum, time since graduation, professional qualifications, experience in work with sporting populations, experience in assessment and management of concussion, and participation in concussion-focused continuing professional development courses.

Sections 2 and 3 used a 5-point Likert scale to investigate participants’ views on 13 factors that may positively or negatively impact their self-efficacy in concussion assessment and management. The assessed factors included practice in both classroom and clinical environments, observation of an educator and peer, verbal encouragement from an educator and peer, physical and emotional relaxation, as well as positive and negative feedback coming from an educator and peer.

In Section 4 of the survey, participants rated their self-efficacy beliefs separately for 19 concussion assessment (8 initial assessment and 11 office assessment) skills, and 13 concussion management (6 postconcussion advice and 7 direct management/rehabilitation) skills, on a scale of 0 to 100, with 0, 50, and 100 representing beliefs of “cannot do at all,” “moderately can do,” and “highly certain can do,” respectively.^3^ Skills considered relevant for all the clinician groups were the initial assessment and postconcussion advice, to reflect Irish EMSPs’ scope of practice in relation to head injuries. Office assessment and direct management/rehabilitation skills were considered relevant for CATs and ChPs. Participants’ current practices in assessment and management of concussion were rated alongside self-efficacy beliefs, using a 0 to 100 scale. The ratings 0, 50, and 100 represented statements of “never use the technique with concussed patients,” “use the technique with half of concussed patients,” and “use the technique with every concussed patient.” Participants were also asked to report whether each of the skills was included in the curriculum of their professional education or a continuing professional development course. Cronbach alpha analysis demonstrated good/excellent internal consistency for self-efficacy (0.95), frequency of use (0.95), and positive (0.91) and negative (0.92) factor scales.

### Procedures

Ethical approval was granted by the Dublin City University Research Ethics Committee and participants completed informed consent before completing the survey. Face validity of the survey was established by 5 multinational concussion experts (2 Irish, 2 American, and 1 Canadian). Each question was rated between 1 and 5 for clarity, comprehensiveness, and appropriateness. All questions with a mean score <4 were modified or removed completely, based on the experts’ recommendations.^23^ The survey was then piloted on 10 healthcare professionals. After review of pilot participant feedback, parts of the survey were removed to avoid repetitive questioning and to decrease time required to complete the survey. The final survey was distributed online on SurveyMonkey and shared with the representatives of Irish governing bodies of allied healthcare professions for distribution among their members. Social media platforms as well as word of mouth were also used to recruit participants. The survey was open from November 2022 to March 2023, and the final survey took a mean of 9 ± 5 minutes to fully complete.

### Statistical Analysis

Data were analyzed in SPSS (Version 27, IBM Corp). Descriptive statistics were calculated for demographic information. Means and standard deviations were also calculated for self-efficacy scores, frequency of skills use, and for the factors impacting self-efficacy. All data, apart from general concussion assessment self-efficacy scores, were non-normally distributed. Spearman Rank Order correlation was used to explore the relationship between skills’ self-efficacy and frequency of their use. Effect sizes were classified as small (*r* = 0.10), medium (*r* = 0.30), and large (*r* = 0.50).^21^ Kruskal-Wallis tests and pairwise comparisons were also used to examine differences in immediate assessment and postconcussion advice self-efficacy and frequency of use among CATs, ChPs, EMSPs, and clinicians holding both CAT and ChP qualifications (CAT/ChPs). Statistical significance was set at *P* < 0.05; however, Bonferroni adjustment was applied to post hoc tests (*P* < 0.01). Effect sizes were classified as small (η^2^ = 0.01), medium (η^2^ = 0.06), or large (η^2^ = 0.14).^21^ One-way analysis of variance with Bonferroni post hoc tests were used to analyze differences in overall concussion assessment self-efficacy scores among CATs, ChPs, and CAT/ChPs. Kruskal-Wallis tests and pairwise comparisons were used to establish differences in concussion assessment frequency of use, concussion management self-efficacy, and frequency of use among CATs, ChPs, and CAT/ChPs. Friedman’s test examined differences in ratings of factors impacting self-efficacy positively and negatively. Effect sizes were classified as small (w = 0.1), medium (w = 0.30), or large (w = 0.50).^21^ Multiple Wilcoxon signed-rank order tests were used to explore the impact of location of practice, type of feedback and source of encouragement, vicarious experience, and feedback on self-efficacy. Bonferroni adjusted alpha level for these tests was *P* < 0.01, and the effect sizes were classified as small (*r* = 0.10), medium (*r* = 0.30), and large (*r* = 0.50).^21^

## Results

### Participant Demographics

In total, 285 allied healthcare professionals’ responses were included in the analysis. There were 68.4% (195) male participants, 31.2% (89) female participants, and 0.4% (1) nonbinary participant, with a mean age of 35.1 ± 10.3 (range, 0-47) years. Postgraduate qualifications were held by 58.2% (166) of practitioners. The majority of participants worked with sporting populations (84.2%, 240), had a mean professional experience of 9.3 ± 8.2 years in sport, and 45.6% ± 31.2% of their patients presented with sporting injuries. Participating professionals assessed or managed 10.7 ± 16.2 concussions annually on average. Table 1 displays participant demographics. Table 2 displays differences in professional engagement across clinician groups.

**Table 1.** Participant demographics

		Mean ± SD	Frequency, % (n)
Primary professional qualification	ChP		33.7 (96)
	EMSP		33.3 (95)
	CAT		27.4 (78)
	CAT/ChP		5.6 (16)
Time from most recent graduation, y		5.93 ± 5.86	
Sporting populations working with	Community athletes (nonprofessional/developmental level)		66 (188)
	Elite athletes (professional/inter-county/national level)		51.6 (147)
	Children (0-10 years old)		43.9 (125)
	Adolescent (11-18 years old)		65.3 (186)
	Adults		76.1 (217)
Participation in concussion-related CPD courses	No		54.7 (156)
	Yes		45.3 (129)
	Number of CPDs attended	2.38 ± 1.46	

CAT, certified athletic therapist; ChP, chartered physiotherapist; CPD, continuous professional development; EMSP, emergency medical services practitioner.

**Table 2.** Differences in professional engagement across practitioner groups

	ChP (n = 96)	EMSP (n = 95)	CAT (n = 78)	CAT/ChP (n = 16)
Working with sporting populations	91.7% (88)	66.3% (63)	93.6% (73)	100% (16)
Community athletes	75.0% (72)	58.5% (55)	64.1% (50)	68.8% (11)
Elite athletes	60.4% (58)	36.8% (35)	57.7% (45)	56.3% (9)
Children	46.9% (45)	48.4% (46)	37.2% (29)	31.3% (5)
Adolescent	67.7% (65)	51.6% (49)	79.5% (62)	62.5% (10)
Adults	79.2% (76)	62.1% (59)	85.9% (67)	93.8% (15)
Engagement in concussion related CPD	67.7% (65)	36.8% (35)	26.9% (21)	50.0% (8)

CAT, certified athletic therapist; ChP, chartered physiotherapist; CPD, continuous professional development; EMSP, emergency medical services practitioner.

### Concussion Assessment and Management Skills

The overall levels of self-efficacy among CATs, ChPs, EMSPs, and CAT/ChPs were 64.5 ± 26.6 for immediate concussion assessment and 56.6 ± 25.4 for postconcussion advice. The highest scores were for concussion symptom checklist (80 ± 28.4) and advice on physical rest (80.1 ± 27.8), while the lowest were for a Child Sport Concussion Assessment Tool (SCAT5) (44.6 ± 41.2) and advice on nutrition (34.1 ± 33.7) (Table 3). Considering all concussion assessment and management skills among CATs, ChPs, and CAT/ChPs, the self-efficacy levels were 51.5 ± 20.1 and 62.1 ± 20.9, respectively. The highest scores were for history/clinical evaluation nonspecific to concussion (86.6 ± 16.2) and advice on physical rest (86.3 ± 20). The lowest were for paper/pencil neuropsychological test (16.7 ± 28.6) and advice on a use of medication (39.2 ± 35.0) (Table 4). A strong positive correlation was observed between clinicians’ self-efficacy and frequency of use of overall concussion assessment (*r* = 0.795; *P* < 0.01) and management (*r* = 0.812; *P* < 0.01), immediate concussion assessment (*r* = 0.728; *P* < 0.01) and advice (*r* = 0.805; *P* < 0.01) skills, as well as each specific skill individually (Tables 3 and 4). Professionals holding both CAT and ChP qualifications had the highest self-efficacy scores for concussion assessment (60.4 ± 18) and management (64.2 ± 17.9) when compared with CATs and ChPs, as well as demonstrated the highest frequency of use of assessment (39.8 ± 17.7) and management (48.4 ± 22.7) skills (Table 5). No significant differences were observed between these clinician groups when considering concussion assessment and management self-efficacy and frequency of use scores. Regarding the immediate concussion assessment, professionals holding both CAT and ChP qualifications had the highest self-efficacy (77 ± 15.9) and frequency of use (65.1 ± 24.3) scores when compared with CATs, ChPs, and EMSPs. They also displayed the highest frequency of postconcussion advice (58.9 ± 22.2), while CATs displayed the highest self-efficacy score for advice (65.7 ± 20.7) (Table 6). Kruskal-Wallis tests revealed significant differences in immediate concussion assessment self-efficacy [H(3) = 40.16; *P* < 0.01, η^2^ = 0.25] and frequency [H(3) = 25.15; *P* < 0.01, η^2^ = 0.16] and postconcussion advice self-efficacy [H(3) = 18.62; *P* < 0.01, η^2^ = .13] and frequency [H(3) = 12.63; *P <* 0.01, η^2^ = 0.09] among clinician groups. Pairwise post hoc comparisons indicated significantly lower immediate concussion assessment self-efficacy and frequency and postconcussion advice self-efficacy and frequency scores of EMSPs when compared with CATs (*P* < 0.01), ChPs (*P* < 0.01), and CATs/ChPs (*P* < 0.01).

**Table 3.** Self-efficacy levels and frequency of use for immediate concussion assessment and postconcussion advice skills among Irish CATs, ChPs, EMSPs, and CAT/ChPs, with correlation coefficients for the relationship between self-efficacy and frequency of use

Immediate Concussion Assessment/Advice Skill	Self-efficacy (0-100), Mean ± SD^*a*^	Frequency of Use With Concussed Patients (0-100), Mean ± SD^*b*^	Correlation Coefficient	*P* Value
Assessment of concussion relevant health history (eg, previous concussions, ADHD, learning difficulties, migraines)	70 ± 28.5	53.4 ± 41.1	*r* = 0.646	<0.01*
History and clinical evaluation nonspecific to concussion (eg, cervical ROM, neck strength, myotomes/dermatomes)	76.7 ± 29.5	63.5 ± 38	*r* = 0.793	<0.01*
Any concussion symptom checklist	80 ± 28.4	60.2 ± 41.4	*r* = 0.694	<0.01*
SAC	54.4 ± 41.1	35.6 ± 39.5	*r* = 0.821	<0.01*
SCAT 5	70.9 ± 36.7	49.1 ± 42.9	*r* = 0.791	<0.01*
Child SCAT5	44.6 ± 41.2	21.8 ± 36.2	*r* = 0.674	<0.01*
Balance measure (eg, BESS)	61.2 ± 39.9	44.3 ± 42.7	*r* = 0.811	<0.01*
Gait measure (eg, timed tandem gait)	58.3 ± 40.3	37.9 ± 40.9	*r* = 0.796	<0.01*
Providing advice on cognitive rest	71 ± 33	59.3 ± 41.8	*r* = 0.738	<0.01*
Providing advice on physical rest	80.1 ± 27.8	67.3 ± 40.2	*r* = 0.727	<0.01*
Providing advice on use of medications	41.1 ± 36.8	31.9 ± 37.3	*r* = 0.857	<0.01*
Providing advice on nutrition	34.1 ± 33.7	22.3 ± 30.2	*r* = 0.819	<0.01*
Providing advice on driving	56.1 ± 38.5	42 ± 40	*r* = 0.813	<0.01*
Providing advice on return to school/learning activities	56.6 ± 36.9	46.6 ± 40	*r* = 0.837	<0.01*

ADHD, attention deficit hyperactivity disorder; BESS, balance error scoring system; CAT, certified athletic therapist; ChP, chartered physiotherapist; EMSP, emergency medical services practitioner; ROM, range of motion; SAC, standard assessment of concussion; SCAT5, Sport Concussion Assessment Tool.

a 0, cannot do at all; 50, moderately certain can do; 100, highly certain can do.

b 0, never use the technique; 50, use the technique with half of concussed athletes; 100, use the technique with every concussed athlete.

* Statistical significance at the 0.01 level.

**Table 4.** Self-efficacy levels and frequency of use for concussion assessment and management relevant skills among Irish CATs, ChPs, and CAT/ChPs, with correlation coefficients for the relationship between self-efficacy and frequency of use

Concussion Assessment/Management Skill	Self-efficacy (0-100), Mean ± SD^*a*^	Frequency of Use With Concussed Patients (0-100), Mean ± SD^*b*^	Correlation Coefficient	*P* Value
Assessment of concussion relevant health history (eg, previous concussions, ADHD, learning difficulties, migraines)	72.4 ± 26.5	55.8 ± 41	*r* = 0.646	<0.01*
History and clinical evaluation non-specific to concussion (eg, cervical ROM, neck strength, myotomes/dermatomes)	86.6 ± 16.2	74.1 ± 31.9	*r* = 0.793	<0.01*
Cervical spine tests (eg, cervical joint-reposition error test, smooth-pursuit neck torsion test)	55 ± 33.3	37.6 ± 34.5	*r* = 0.831	<0.01*
Cranial nerve examination	49.4 ± 32.6	31.6 ± 36.6	*r* = 0.819	<0.01*
Any concussion symptom checklist	86.4 ± 22.4	69.6 ± 39.1	*r* = 0.694	<0.01*
SAC	58.1 ± 40.6	37.8 ± 40.2	*r* = 0.821	<0.01*
SCAT5	82.3 ± 28.1	59.4 ± 41.1	*r* = 0.791	<0.01*
Child SCAT5	52.7 ± 40.9	25.5 ± 39.1	*r* = 0.674	<0.01*
Balance measure (eg, BESS)	76.9 ± 29.8	56.8 ± 40.9	*r* = 0.811	<0.01*
Gait measure (eg, timed tandem gait)	71.3 ± 34.8	47 ± 41.6	*r* = 0.796	<0.01*
Vestibular/ocular motor test (eg, VOMS)	57.8 ± 39.7	44.4 ± 42.9	*r* = 0.875	<0.01*
King-Devick Test	20.8 ± 33.5	5.4 ± 16.3	*r* = 0.602	<0.01*
Paper/pencil neuropsychological test (eg, the Symbol Digit Modalities Test)	16.7 ± 28.6	7.1 ± 19.6	*r* = 0.708	<0.01*
Computerized neuropsychological test (eg, ImPACT)	23.3 ± 34.6	10.1 ± 25.4	*r* = 0.704	<0.01*
Reaction time testing not included in computerized neuropsychological testing (eg, ruler drop test)	26.8 ± 35.9	9.4 ± 21.7	*r* = 0.687	<0.01*
Aerobic exercise tolerance test (eg, Buffalo Concussion Treadmill Test)	42.1 ± 40.6	18.7 ± 30.7	*r* = 0.723	<0.01*
Mood, anxiety or depression assessment (eg, Brief Symptom Inventory-18)	41.1 ± 39.9	23.4 ± 34.6	*r* = 0.746	<0.01*
Sleep quality and quantity measure (eg, Pittsburgh Sleep Quality Index)	39.3 ± 39.8	14.8 ± 27.7	*r* = 0.715	<0.01*
Migraine assessment (eg, The Migraine Disability Assessment)	26.1 ± 3	10.9 ± 24.3	*r* = 0.724	<0.01*
Providing advice on cognitive rest	79.3 ± 25.7	66.3 ± 39.7	*r* = 0.738	<0.01*
Providing advice on physical rest	86.3 ± 20	74.7 ± 36.7	*r* = 0.727	<0.01*
Providing advice on use of medications	39.2 ± 35	28.9 ± 34.8	*r* = 0.857	<0.01*
Providing advice on nutrition	39.2 ± 32.4	24.8 ± 30.1	*r* = 0.819	<0.01*
Providing advice on driving	60.6 ± 36.1	45.8 ± 39.6	*r* = 0.813	<0.01*
Providing advice on return to school/learning activities	66.5 ± 30.5	55.1 ± 38.2	*r* = 0.837	<0.01*
Prescription of aerobic exercise	78.2 ± 26.4	69.9 ± 37.3	*r* = 0.838	<0.01*
Return to play progression (as per consensus statements eg, graduated stepwise progression)	77.5 ± 28.3	65.7 ± 39.1	*r* = 0.823	<0.01*
Balance training	75.4 ± 28.6	58 ± 37.8	*r* = 0.852	<0.01*
Cervical spine rehabilitation	69.8 ± 31.8	50.7 ± 37.6	*r* = 0.835	<0.01*
Treatment of chronic headache	39.6 ± 32.4	24.5 ± 30.9	*r* = 0.793	<0.01*
Vestibular/ocular motor rehabilitation	40.7 ± 37.8	29.4 ± 36.1	*r* = 0.889	<0.01*
Referral to a specialist (eg, optometrist, vestibular specialist, psychologist)	60.1 ± 37.4	25.8 ± 31.8	*r* = 0.808	<0.01*

ADHD, attention deficit hyperactivity disorder; BESS, balance error scoring system; CAT, certified athletic therapist; ChP, chartered physiotherapist; EMSP, emergency medical services practitioner; ROM, range of motion; SAC, standard assessment of concussion; SCAT5, Sport Concussion Assessment Tool; VOMS, vestibular/ocular motor screening.

a 0, cannot do at all; 50, moderately certain can do; 100, highly certain can do.

b 0, never use the technique; 50, use the technique with half of concussed athletes; 100, use the technique with every concussed athlete.

* Statistical significance at the 0.01 level.

**Table 5.** Overall concussion assessment and management skills’ self-efficacy levels and frequency of use across professional groups

	Concussion Assessment Skills		Concussion Management Skills	
	Self-efficacy (0-100), Mean ± SD^*a*^	Frequency of Use (0-100), Mean ± SD^*b*^	Self-efficacy (0-100), Mean ± SD^*a*^	Frequency of Use (0-100), Mean ± SD^*b*^
CAT	52.1 ± 19.8	34.3 ± 22.1	61.5 ± 22.1	47.3 ± 25
ChP	49.7 ± 20.7	30.9 ± 19.3	62.3 ± 20.7	45.8 ± 25.4
CAT/ChP	60.4 ± 18	39.8 ± 17.7	64.2 ± 17.9	48.4 ± 22.7

CAT, certified athletic therapist; ChP, chartered physiotherapist.

a 0, cannot do at all; 50, moderately certain can do; 100, highly certain can do.

b 0, never use the technique; 50, use the technique with half of concussed athletes; 100, use the technique with every concussed athlete.

**Table 6.** Immediate concussion assessment and post-concussion advice skills’ self-efficacy levels and frequency of use across professional groups

	Immediate Concussion Assessment Skills		Postconcussion Advice Skills	
	Self-efficacy (0-100), Mean ± SD^*a*^	Frequency of Use (0-100), Mean ± SD^*b*^	Self-efficacy (0-100), Mean ± SD^*a*^	Frequency of Use (0-100), Mean ± SD^*b*^
CAT	73.3 ± 20.1	54.7 ± 28.3	65.7 ± 20.7	52.7 ± 28.3
ChP	72.4 ± 22.6	50 ± 29.9	58.6 ± 22	45 ± 28.4
CAT/ChP	77 ± 15.9	39.8 ± 17.7	61.4 ± 22.5	58.9 ± 22.2
EMSP	40.8 ± 26	26.2 ± 23.7	40.4 ± 29.3	31.9 ± 30.7

CAT, certified athletic therapist; ChP, chartered physiotherapist; EMSP, emergency medical services practitioner.

a 0, cannot do at all; 50, moderately certain can do; 100, highly certain can do.

b 0, never use the technique; 50, use the technique with half of concussed athletes; 100, use the technique with every concussed athlete.

### Factors Impacting Self-efficacy

**Figure 1.**
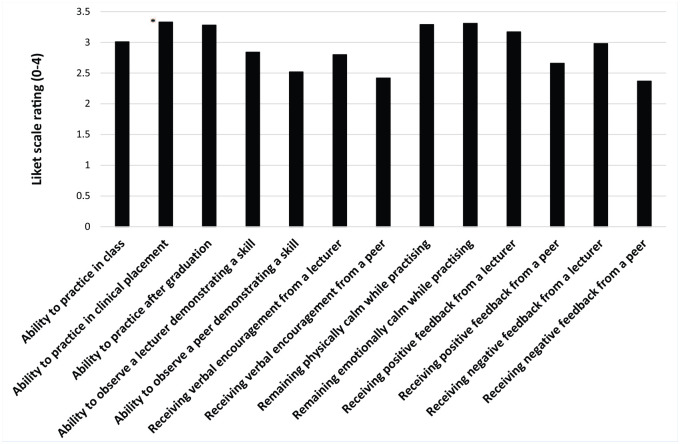
Distribution of scores for factors having positive impact of self-efficacy in concussion assessment and management.

**Figure 2.**
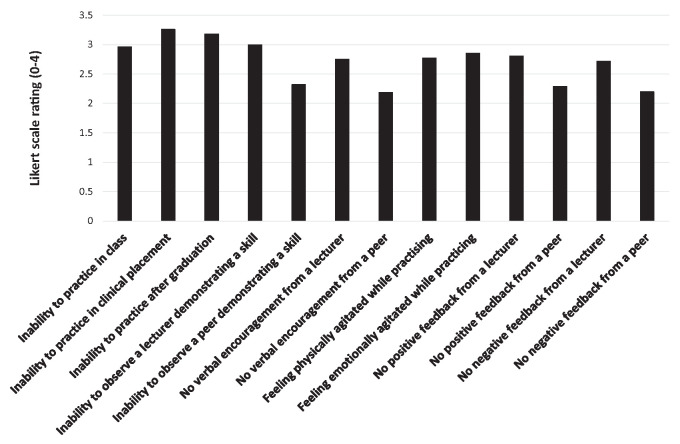
Distribution of scores for factors having negative impact of self-efficacy in concussion assessment and management.

Ability to practice skills during clinical placement (3.3 ± 0.9) and remaining emotionally (3.3 ± 0.8) and physically (3.3 ± 0.8) calm while practicing the skills (Figure 1) had the greatest positive impact on clinicians’ self-efficacy. The greatest negative impact was the inability to practice skills in clinical placement (3.3 ± 1.0), independently after graduation (3.2 ± 1.0), or to observe a lecturer demonstrating the skills (3 ± 1.1) (Figure 2). Significant differences were found between factors positively [χ^2^(12) = 514.77; *P* < 0.01; W = 0.21] and negatively [χ^2^(12) = 445.51; *P* < 0.01; W = 0.19] impacting self-efficacy. Ability to practice skills in clinical placement (Z = -6.48; *P* < 0.01; *r* = 0.42) and independently after graduation (Z = -4.44; *P* < 0.01; *r* = 0.28) had significantly higher positive impact on professionals’ self-efficacy when compared with practice in a practical class. Ability to observe the lecturer demonstrating a skill (Z = -6.26; *P* < 0.01; *r* = 0.39) as well as receiving their verbal encouragement (Z = -6.96; *P* < 0.01; *r* = 0.44) had significantly greater positive impact on self-efficacy than observation of a peer or peer encouragement. Lecturer (Z = -3.80; *P* < 0.01; *r* = 0.24), and peer (Z = -4.75; *P* < 0.01; *r* = 0.30) positive feedback impacted self-efficacy significantly more compared with negative feedback. Both positive (Z = -8.10; *P* < 0.01; *r* = 0.51) and negative (Z = -8.31; *P* < 0.01; *r* = 0.52) feedback from a lecturer were rated significantly higher than either type of feedback from a peer. Inability to practice skills in clinical placement had a significantly higher negative impact on practitioner’s self-efficacy than inability to practice them in a practical class (Z = -4.87; *P* < 0.01; *r* = 0.32). Inability to observe the lecturer demonstrating a skill (Z = -8.65; *P* < 0.01; *r* = 0.55) and not receiving verbal encouragement (Z = -7.29; *P* < 0.01; *r* = 0.47) had significantly greater negative impact on self-efficacy than inability to observe a peer or not receiving their encouragement. Lack of both positive (Z = -8.12; *P* < 0.01; *r* = 0.52) and negative (Z = -7.02; *P* < 0.01; *r* = 0.45) feedback from a lecturer had a significantly higher negative impact on self-efficacy than lack of peer feedback.

## Discussion

This study aimed to explore the concept of self-efficacy in concussion assessment and management among Irish allied healthcare professionals, to investigate the relationship between their self-efficacy and clinical practices, and to establish the factors that impact development of their self-efficacy.

The general levels of self-efficacy were moderate for immediate concussion assessment, postconcussion advice, overall concussion assessment and management. American ATs’ self-efficacy was higher than Irish clinicians for concussion assessment (60.3 ± 14.5 vs 51.5 ± 20.1); however, efficacy was lower for management (55.3 ± 14.1 vs 62.1 ± 20.9).^61^ A possible explanation of these results is that American ATs may get more exposure to concussions and their assessment (14 vs 10 annually), compared with Irish clinicians, who may engage with greater treatment/rehabilitation of concussed patients. The differences in concussion management self-efficacy may also result from recent advances in concussion treatment/rehabilitation,^27,60^ allowing our participants to broaden their concussion management skill set and become more confident at it. No other research has examined clinicians’ self-efficacy in balance training, cervical spine rehabilitation, or treatment of chronic headaches.

Considering specific skills, the highest scores were for concussion symptom checklist and history/clinical evaluation nonspecific to concussion, both in the analysis of all Irish clinicians’ immediate assessment skill, and among CATs, ChPs, and CAT/ChPs, considering all the assessment skills. This is in line with the past research on American ATs; however, their scores were higher (95 ± 7.5 vs 80 ± 28.4/86.4 ± 22.4 and 92.2 ± 12.5 vs 76.7 ± 29.5/86.6 ± 16.2).^61^ Concussion symptom checklists have been used in concussion assessment for many years,^2^ and the majority of American ATs are examined on this skill during professional education.^68^ Moreover, a high number of American sports medicine clinicians (85.3%),^7^ ATs (86.7%),^37^ and Irish CATs (93.1%) had indicated its use in clinical practice.^36^ Practice is suggested to be the strongest self-efficacy impacting factor,^59^ so the high scores for this skill are understandable. Similarly, the high scores for the history assessment may be linked to its nonspecific nature, whereby clinicians use this skill with patients regularly, developing self-efficacy through frequent use. The lowest score, considering the immediate assessment skills of all Irish clinicians, was for a Child SCAT5 (44.6 ± 41.2). Less than half of our participants (31.3%-46.9%, depending on professional group) worked with children, thus opportunity to practice may be scarce. Moreover, lack of education on child concussion has been reported among emergency medical personnel globally.^64^ Considering the overall assessment, Irish CATs, ChPs, and CAT/ChPs scored the lowest for paper/pencil neuropsychological test (16.7 ± 28.6) and King-Devick Test (20.8 ± 33.5), similar to American ATs (5.8 ± 21.1 and 12.7 ± 32.5).^61^ A practical application of paper/pencil neuropsychological testing might not be included in the curriculum of AT education, as only 10.33% of ATs reported using it in practice, and 78.8% believed they should be trained in its administration.^37^ Conversely, computerized neuropsychological assessment is taught in 89.4% of athletic training programmes,^68^ which corresponds with the high scores (77.6 ± 36.2) of self-efficacy for this skill among American ATs.^61^ Low self-efficacy scores of paper/pencil neuropsychological testing among our participants are in line with its very low (5.3%) use among Irish CATs.^36^ This, however, cannot be justified by a higher use of computerized neuropsychological assessment as the levels of both were identical (5.3%).^36^ Interestingly, computerized neuropsychological assessment was the third lowest scoring (23.3 ± 34.6) skill among our participants, possibly due to the costs associated with it. The King-Devick test is a relatively new diagnostic tool, so clinicians might not be experienced in its use.^61^ This is in line with recent evidence suggesting that only 26.6% of American educational institutions provide athletic therapy students with hands-on experience in the use of the King-Devick test,^68^ and only 3.2% of American sports medicine clinicians administer it on concussed collegiate athletes.^7^ Importantly, the King-Devick test requires a baseline assessment,^33^ particularly for athletes with learning and attentional disabilities,^45^ and there is a cost associated with its use.^61^ Moreover, some concern has been raised in the literature over its high false-positive rates.^24,26^ It is possible that clinicians use different tools to assess ocular function.

Considering concussion management, advice on physical and cognitive rest were rated the highest in the analysis of all clinicians’ advice skills and the overall management skills of CATs, ChPs, and CAT/ChPs. The latter also scored highly for prescription of aerobic exercise and RTP progression, in line with American ATs, who reported very high self-efficacy for stepwise progression and homecare instructions.^61^ The importance of relative physical and cognitive rest in concussion recovery has been emphasized for many years now, with clear guidelines for safe RTP available for clinicians.^42^ In Ireland, 86.8% of CATs reported using these guidelines in their clinical practice,^36^ which corresponds with the self-efficacy scores of our participants, and all major sporting bodies in Ireland have adopted RTP protocols following a concussion.

The lowest scores were for advice on nutrition and the use of medication, among all Irish clinicians (34.1 ± 33.7 and 41.1 ± 36.8) and among CATs, ChPs, and CAT/ChPs (39.2 ± 32.4 and 39.2 ± 35). The evidence on the potential influence of nutrition on recovery from concussion has started to emerge in the literature only recently,^28,69^ so clinicians might not be up to date with these recent publications. They also may not be embedded into clinicians’ education curriculums. Deficits in nutrition-related knowledge and confidence were reported previously among Irish doctors.^10^ Recommendations on the use of pharmacological treatment in concussion have been available for over a decade now^44^; however, Irish clinicians might not be educated on this topic. Although EMSPs and CATs are authorized to legally administer certain medication,^14-17^ these may not be applicable for concussion. American ATs scored the lowest (17.6 ± 31.7) on vestibular/ocular motor therapy, apart from tests repeatedly included as assessment and management tools (e.g., the paper/pencil neuropsychological test and King-Devick test).^61^ Our participants also presented low self-efficacy for this skill (40.7 ± 37.8). Assessment of vestibular/oculomotor function in concussed athletes has been introduced quite recently, hence rehabilitation of these disorders is a relatively new skill.^61^ Less than half (48.6%) of American physical therapists reported using it in clinical practice,^72^ which may explain the low self-efficacy scores.

Our comparison of Irish clinician groups indicated significantly lower self-efficacy and frequency of use of the immediate concussion assessment (40.8 ± 26 and 26.2 ± 23.7) and postconcussion advice (40.4 ± 29.3 and 85 ± 30.7) skills among EMSPs, when compared with other professional groups. Until recently, paramedics and advanced paramedics in Ireland have been responsible solely for emergency patient care and life-support services^6,53,71^; however, all EMSPs in our study reported assessing/managing concussion as a part of their practice. The education and training standards approved by the Pre-hospital Emergency Care Council (PHECC) in 2014 include head injuries at all 3 levels of National Qualification in Emergency Technology.^54-56^ However, assessment/management of concussion specifically is solely included at the Paramedic level, with the only specific skill listed being Maddocks questions.^56^ This corresponds with past literature indicating that, although assessment and management of traumatic brain injury is within the Canadian paramedics’ scope of practice, concussion is not included in sufficient detail in the curriculum of their education, and they may not be up-to-date with the recent evidence regarding concussion care.^65^ Considering that 66.3% of EMSPs in this study reported working with sporting populations and 36.8% participated in concussion-related CPD, it appears crucial to revise the PHECC approved education and training standards.

The highest level of self-efficacy in both concussion assessment (60.4 ± 18) and management (64.2 ± 17.9) demonstrated clinicians holding both CAT and ChP qualifications. No comparative data exist in the literature; however, there is some evidence that clinicians holding postgraduate qualifications are more likely to comply with gold standard concussion care recommendations.^36,37^ Through a longer education pathway, professionals holding dual qualifications may have had a greater exposure to concussion assessment and management education and more opportunities to practice concussion-related skills under supervision. Past research emphasizes the impact of practice and role models on development of self-efficacy.^66^ In addition, all professionals holding dual qualifications reported working with sporting populations, as opposed to the other professional groups (66.3%-93.6% working with sporting populations). This clinical experience could potentially allow them to assess/manage more concussions and consequently develop higher self-efficacy levels. Importantly, however, the number of clinicians holding dual qualifications in our study was quite low (n = 16) when compared with ChPs (n = 96) and CATs (n = 78), which may have impacted our findings. We observed strong positive correlation between self-efficacy levels and frequency of use of each concussion-related skill. It has been advocated that practice impacts development of self-efficacy, while the level of self-efficacy in a particular skill impacts the likelihood of performing that skill.^62^ Although no research investigated the relationship of self-efficacy and practice in relation to concussion assessment and management, literature independently exploring concussion-related self-efficacy and clinical practices seem to support this trend.^37,61^ Our study confirmed that concussion related self-efficacy scores correlate with frequency of skill use. Thus, ensuring sufficient time and opportunities to practice during education might be crucial to the development of self-efficacy, which, in turn, may allow clinicians to use the learnt skills efficaciously after graduation, and further reinforce their self-efficacy belief.The views of our participants support this. We found that the ability to practice skills during clinical placement influences clinician’s self-efficacy perceptions the most. Past literature suggests mastery experience as the strongest self-efficacy impacting factor,^59^ and healthcare-related research confirms this, proposing practice in a clinical environment and observation of role models as other crucial factors.^18,29,66,72^ Considering the nature of practice during clinical placement, where mastery experience takes place in a clinical environment and alongside role modelling, a high rating of this experience in comparison to practice in a practical class or independently after graduation seems understandable. Notably, our participants rated independent practice after graduation significantly higher than practice in a classroom. Thus, mastery experience in a clinical environment may impact self-efficacy much more strongly than a mastery experience with role modelling in a classroom setting. Literature suggests that the impact of role models on self-efficacy might be 3-fold, through skill demonstration, provision of feedback, and encouragement to further practice.^66^ Importantly, however, all the above should come from a “significant other,” ie, a person viewed as knowledgeable and credible.^11^ Our results demonstrated similar beliefs among clinicians. Interaction with a lecturer was rated significantly higher than that with a peer, with educator’s positive feedback having particularly strong impact on development of self-efficacy. Lack of physical and emotional arousal while practicing the skills was reported to have a great impact on our participants’ self-efficacy. Although literature suggests this is the least influencing factor among those listed in Bandura’s self-efficacy model,^59^ it is particularly important in relation to physically or emotionally demanding activities.^38^ Hence bodily reactions may be an important source of personal efficacy information in relation to concussion-care, which is challenging for clinicians.^42^

## Limitations

Convenience sampling was used, which likely led to selection bias. It is likely that clinicians with more interest in concussion or higher concussion-related self-efficacy levels decided to participate in the study. We were unable to calculate the response rate for our survey, as the total number of clinicians involved in team sports athlete care in Ireland is unknown. We have used a self-report survey, which may have led to inaccuracy of some responses. Although all participants reported to be professionally involved in concussion assessment and/or management, we cannot be sure that all engage equally in both aspects of concussion care and get a chance to utilize all the skills in clinical practice. The potential differences in head injury related protocols implemented at each participant’s specific work-setting were also not considered. Hence the differences in their self-efficacy level might be linked to the scope of their professional practice. An investigation of practice-specific skills self-efficacy and exploration of its impact on clinical practice would be warranted, as well as investigation of any variations in those, depending on sport level, type, and requirements put upon clinicians regarding their continuous professional development. Our study was limited to clinicians who most commonly work in a team sports setting in Ireland. Thus, examining concussion-related self-efficacy among sports medicine physicians, neurologists, and other healthcare professionals involved in concussion care would be beneficial.

## Conclusion

Concussed patients in Ireland receive care from clinicians who feel only moderately efficacious about delivery of gold-standard concussion-related skills. Irish clinicians reported high self-efficacy in skills that are well established in clinical practice, and for which instructive and accessible tools exist. However, they did not feel efficacious in neuropsychological assessment and skills recently introduced to clinical practice. The newly developed SCAT6/SCOAT6 tools might facilitate growth of concussion-related self-efficacy among the Irish clinicians.^51,52^ EMSPs were significantly less efficacious in the immediate concussion assessment and postconcussion advice than any other professional group. Clinicians who used concussion-relevant skills more frequently in practice displayed higher levels of self-efficacy for these skills. Self-efficacy in concussion assessment and management can be enhanced through practice in a clinical environment and through experiencing composure while practicing.

## Supplemental Material

sj-pdf-1-sph-10.1177_19417381241287209 – Supplemental material for Concussion Assessment and Management Self-efficacy Among Irish Clinicians Supplemental material, sj-pdf-1-sph-10.1177_19417381241287209 for Concussion Assessment and Management Self-efficacy Among Irish Clinicians by Anna P. Postawa, Siobhán O’Connor and Enda F. Whyte in Sports Health

## References

[1] Ajzen I. The theory of planned behavior. Organ Behav Hum Decis Process. 1991;50(2):179-211.

[2] Aubry M Cantu R Dvorak J , et al. Summary and agreement statement of the first International Conference on Concussion in Sport, Vienna 2001. Br J Sports Med. 2002;36(1):6-7. 10.1136/bjsm.36.1.6 PMC1724447 11867482

[3] Bandura A . Guide to the construction of self-efficacy scales. In: Pajares F Urdan T , eds. Self-Efficacy Beliefs of Adolescents. Charlotte, NC: Information Age Publishing; 2006:307-337.

[4] Bandura A . Social cognitive theory. In: Vasta R ed. Annals of Child Development, Vol 6. Six Theories of Child Development. Greenwich, CT: JAI Press; 1989:1-60.

[5] Bandura A Freeman WH Lightsey R. Self-efficacy: the exercise of control. J Cogn Psychother. 1997;13(2):158-166.

[6] Barry T Batt A Agarwal G Booker M Casey M McCombe G. Potential for paramedic roles in Irish general practice: a qualitative study of stakeholder’s perspectives. HRB Open Res. 2022; 5:40. 36072818 10.12688/hrbopenres.13545.1 PMC9418754

[7] Baugh CM Kroshus E Stamm JM Daneshvar DH Pepin MJ Meehan WP III . Clinical practices in collegiate concussion management. Am J Sports Med. 2016;44(6):1391-1399. 27037282 10.1177/0363546516635639 PMC4891296

[8] Boland K Allen K James A Dunne C. Starving for knowledge. Addressing the place of clinical nutrition within undergraduate and postgraduate clinical training: a national survey of practicing clinicians. Clin Nutr ESPEN. 2022; 52:377-380. 36513479 10.1016/j.clnesp.2022.09.020

[9] Black AM Yeates KO Babul S Nettel-Aguirre A Emery CA. Association between concussion education and concussion knowledge, beliefs and behaviours among youth ice hockey parents and coaches: a cross-sectional study. BMJ Open. 2020;10(8):e038166. 10.1136/bmjopen-2020-038166 PMC7445332 32830117

[10] Boggild M Tator CH. Concussion knowledge among medical students and neurology/neurosurgery residents. Can J Neurol Sci. 2012;39(3):361-368. 22547519 10.1017/s0317167100013524

[11] Bong M Skaalvik EM. Academic self-concept and self-efficacy: how different are they really? Educ Psychol Rev. 2003;15(1):1-40.

[12] Buckley TA Burdette G Kelly K. Concussion-management practice patterns of National Collegiate Athletic Association Division II and III athletic trainers: how the other half lives. J Athl Train. 2015;50(8):879-888. 26196701 10.4085/1062-6050-50.7.04 PMC4629946

[13] Caccese JB Eckner JT Franco-MacKendrick L , et al. Interpreting clinical reaction time change and recovery after concussion: a baseline versus norm- based cutoff score comparison. J Athl Train. 2021;56(8):851-859. 34375406 10.4085/1062-6050-457-20 PMC8359707

[14] Carney R . PHECC Clinical practice guidelines, advanced paramedic. https://phecc.sharepoint.com/:b:/s/ClinicalPractice/EdRPLEi0z9pBkYynDX4UujkB8xMt5dhjJsAglHLyisNoNg?e=g9fMyI(Accessed 15 June 2023).

[15] Carney R . PHECC Clinical practice guidelines, emergency first responder. https://phecc.sharepoint.com/:b:/s/ClinicalPractice/EVPBTpNbUghBmJdMOS8MYS0BkqXudJjF7jqGKjkoOki4KA?e=yO1ijk (Accessed 15 June 2023).

[16] Carney R . PHECC Clinical practice guidelines, emergency medical technician. https://phecc.sharepoint.com/:b:/s/ClinicalPractice/ERoucVqagKdNnlcQltv44QUB_loMmCs8C5hmijptfpYeWQ?e=Bn8Nra (Accessed 15 June 2023).

[17] Carney R . PHECC Clinical practice guidelines, paramedic. https://phecc.sharepoint.com/:b:/s/ClinicalPractice/EaKMqj6giDhKi8PFuGbRcUkBTgJIPRHx73N9TjCOKa_Exw?e=N62v1J (Accessed 15 June 2023).

[18] Carson JAS Gillham MB Kirk LM Reddy ST Battles JB . Enhancing self-efficacy and patient care with cardiovascular nutrition education. Am J Prev Med. 2002;23(4):296-302. 12406484 10.1016/s0749-3797(02)00518-4

[19] Cheever K Kawata K Tierney R Galgon A. Cervical injury assessments for concussion evaluation: a review. J Athl Train. 2016;51(12):1037-1044. 27835042 10.4085/1062-6050-51.12.15 PMC5264559

[20] Clugston JR Houck ZM Asken BM , et al. Relationship between the King-Devick test and commonly used concussion tests at baseline. J Athl Train. 2019;54(12):1247-1253. 31584854 10.4085/1062-6050-455-18 PMC6922559

[21] Cohen J. Statistical Power Analysis for the Behavioral Sciences. 2nd ed. Mahwah, NJ: Lawrence Erlbaum Associates; 1988.

[22] Crilly P Patel N Ogunrinde A Berko D Kayyali R. Community pharmacists’ involvement in research in the United Kingdom. Pharmacy. 2017;5(4):48. 28970460 10.3390/pharmacy5030048 PMC5622360

[23] Devereux F Whyte E Issartel J Belton S O’Connor S. Current practices, willingness and perceived ability to implement an injury prevention exercise program among post-primary physical education teachers. J Sch Health. 2022;93(1):25-33. 36029135 10.1111/josh.13242 PMC10087731

[24] Donaworth MA Grandhi RK Logan K , et al. Is current medical education adequately preparing future physicians to manage concussion: an initial evaluation. Phys Sportsmed. 2016;44(1):1-7. 26758683 10.1080/00913847.2016.1135039

[25] DiCerchio LC Oldham JR Glutting JJ Kaminski TW Buckley TA. Repeat administration, but not fatigue, adversely affects King-Devick test performance. Athl Train Sport Health Care. 2021;13(6):e395-e401.

[26] Eddy R Goetschius J Hertel J Resch J. Test-retest reliability and the effects of exercise on the King-Devick test. Clin J Sport Med. 2020;30(3):239-244. 32341291 10.1097/JSM.0000000000000586

[27] Esterov D Thomas A Weiss K. Osteopathic manipulative medicine in the management of headaches associated with postconcussion syndrome. J Osteopath Med. 2021;121(7):651-656. 33831981 10.1515/jom-2020-0035

[28] Finnegan E Daly E Pearce AJ Ryan L. Nutritional interventions to support acute mTBI recovery. Front Nutr. 2022;9:977728. 36313085 10.3389/fnut.2022.977728 PMC9614271

[29] Ford-Gilboe M Laschinger HS Laforet-Fliesser Y Ward-Griffin C Foran S. The effect of a clinical practicum on undergraduate nursing students’ self-efficacy for community-based family nursing practice. J Nurs Educ. 1997;36(5):212-219. 9145339 10.3928/0148-4834-19970501-06

[30] Gardner N Heron N. A scoping review: mapping the evidence for undergraduate concussion education and proposing the content for medical student concussion teaching. Int J Environ Res Public Health. 2022;19(7):4328. 35410008 10.3390/ijerph19074328 PMC8998836

[31] Graham RF van Rassel CR Burma JS , et al. Concurrent validity of a stationary cycling test and the Buffalo concussion treadmill test in adults with concussion. J Athl Train. 2021;56(12):1292-1299. 34911073 10.4085/1062-6050-0003.21 PMC8675311

[32] Haider MN Leddy JJ John G Tiso M Karl A. Concussion management knowledge among residents and students and how to improve it. Concussion. 2017;2(3):CNC40. 10.2217/cnc-2017-0001 PMC6093773 30202581

[33] Howitt S Brommer R Fowler J Gerwing L Payne J DeGraauw C. The utility of the King-Devick test as a sideline assessment tool for sport-related concussions: a narrative review. J Can Chiropr Assoc. 2016;60(4):322-329. 28065993 PMC5178017

[34] Ishii R Schwedt TJ Trivedi M , et al. Mild traumatic brain injury affects the features of migraine. J Headache Pain. 2021;22(1):80. 34294026 10.1186/s10194-021-01291-x PMC8296591

[35] Jervis CG Adams SA Fawkner S Griffin SA. Concussion education in medical students studying in Scotland: an assessment of knowledge and future needs. Brain Inj. 2022;36(9):1196-1203. 35996323 10.1080/02699052.2022.2115139

[36] Lempke LB Bergeron G O’Connor S Lynall RC Resch JE Walton SR. Concussion assessment and management practices among Irish and Canadian athletic therapists: an international perspective. J Athl Train. 2023;58(4):293-304. 35724361 10.4085/1062-6050-0097.22 PMC11215646

[37] Lempke LB Schmidt JD Lynall RC. Athletic trainers’ concussion-assessment and concussion-management practices: an update. J Athl Train. 2020;55(1):17-26. 31855075 10.4085/1062-6050-322-18 PMC6961637

[38] Lewis M Weight E Hendricks K. Teaching methods that foster self-efficacy belief: perceptions of collegiate musicians from the United States. Psychol Music. 2022;50(3):878-894.

[39] Mani A Dastgheib SA Chanor A Khalili H Ahmadzadeh L Ahmadi J. Sleep quality among patients with mild traumatic brain injury: a cross-sectional study. Bull Emerg Trauma. 2015;3(3):93-96. 27162910 PMC4771248

[40] Mathieu F Ellis MJ Tator CH. Concussion education in Canadian medical schools: a 5-year follow-up survey. BMC Med Educ. 2018;18(1):316. 30572879 10.1186/s12909-018-1416-7 PMC6302298

[41] Maxtone S Bishop M Chapple C Tumilty S Quinn D Kennedy E. Physiotherapist involvement in concussion services in New Zealand: a national survey. N Z J Physiother. 2020;48(2):70-79.

[42] McCrory P Meeuwisse W Dvořák J , et al. Consensus statement on concussion in sport - the 5th international conference on concussion in sport held in Berlin, October 2016. Br J Sports Med. 2017;51(11):838-847. 10.1136/bjsports-2017-097699 28446457

[43] McGrann P Keating L. A survey of chartered physiotherapists knowledge and current clinical practice regarding concussion in sport. R Coll Surg Irel Student Med J. 2012;5(1):33-38.

[44] Meehan WP III . Medical therapies for concussion. Clin J Sport Med. 2011;30(1):115-24, ix. 10.1016/j.csm.2010.08.003 PMC3359788 21074086

[45] Mrazik M Naidu D Borza C Kobitowich T Shergill S. King Devick computerized neurocognitive test scores in professional football players with learning and attentional disabilities. J Neurol Sci. 2019;399:140-143. 30807981 10.1016/j.jns.2019.02.020

[46] O’Connor S Geaney D Whyte EF Kontos AP O’Halloran PJ Beidler E. Perceptions of concussion and associated anxiety in Irish collegiate athletes. Sports Health. 2023;15(2):199-209. 36366782 10.1177/19417381221134103 PMC9950993

[47] Paddack M DeWolf R Covassin T Kontos A. Policies, procedures, and practices regarding sport-related concussion in community college athletes. J Athl Train. 2016;51(1):82-88. 26765512 10.4085/1062-6050-51.2.01 PMC4851134

[48] Pagnotta KD Mazerolle SM Yabor TM Salvatore AC Casa DJ. Self-perceived educational preparedness of entry-level athletic trainers regarding preventing sudden death in sport. Athl Train Educ J. 2013;8(3):48-57.

[49] Pajares F. Self-efficacy beliefs in academic settings. Rev Educ Res. 1996;66(4):543-578.

[50] Paloncy KA Georges L Liggett AJ. A high-fidelity simulation is effective in improving athletic training students’ self-efficacy with emergency cardiovascular care skills. Athl Train Educ J. 2019;14(2):108-116.

[51] Patricios JS Schneider KJ Dvorak J Ahmed OH , et al. Consensus statement on concussion in sport: the 6th International Conference on Concussion in Sport - Amsterdam, October 2022. Br J Sports Med. 2023;57:695-711. 10.1136/bjsports-2023-106898 37316210

[52] Patricios JS Davis GA Ahmed OH , et al. Introducing the Sport Concussion Office Assessment Tool 6 (SCOAT6). 2023;57(12):648-650. 10.1136/bjsports-2023-106860 37316211

[53] Pre-Hospital Emergency Care Council. Pre-hospital emergency care and scope of practice. https://www.phecit.ie/Images/PHECC/Public and Patients/PUB041 - Pre-Hospital Emergency Care and Scope of Practice.pdf (Accessed 23 May 2023).

[54] Pre-Hospital Emergency Care Council. Advanced paramedic education and training standard. https://www.phecit.ie/Images/PHECC/Career%20and%20Education/Education%20Standards/STN016%20Advanced%20Paramedic%20Education%20and%20Training%20Standard-V1.pdf (Accessed 15 June 2023).

[55] Pre-Hospital Emergency Care Council. Emergency medical technician education and training standard-V1. https://www.phecit.ie/Images/PHECC/Career%20and%20Education/Education%20Standards/STN014%20%20Emergency%20Medical%20Technician%20Education%20and%20Training%20Standard-V1.pdf.

[56] Pre-Hospital Emergency Care Council. Paramedic education and training standard. https://www.phecit.ie/Images/PHECC/Career%20and%20Education/Education%20Standards/STN015%20-Paramedic%20Education%20and%20Training%20Standard-V1.pdf.

[57] Quatman-Yates CC Hunter-Giordano A Shimamura KK , et al. Physical therapy evaluation and treatment after concussion/mild traumatic brain injury. J Orthop Sports Phys Ther. 2020;50(4):CPG1-CPG73. 10.2519/jospt.2020.0301 32241234

[58] Rashid H Mishra S Dobbin N. Management of sport-related concussion in emergency departments in England: a multi-center study. Brain Inj. 2021;35(9):1035-1042. 34288793 10.1080/02699052.2021.1945146

[59] Richardson A. Developing self-efficacy in the physics classroom through hands-on projects. AIP Conf Proc. 2019;2109(1):120004.

[60] Rytter HM Westenbaek K Henriksen H Christiansen P Humle F. Specialized interdisciplinary rehabilitation reduces persistent post-concussive symptoms: a randomized clinical trial. Brain Inj. 2019;33(3):266-281. 30500267 10.1080/02699052.2018.1552022

[61] Savage JL Covassin T. The self-efficacy of certified athletic trainers in assessing and managing sport-related concussions. J Athl Train. 2018;53(10):983-989. 30311504 10.4085/1062-6050-394-17 PMC6263082

[62] Schunk DH. Self-efficacy and classroom learning. Psychol Sch. 1985;22(2):208-223.

[63] Schunk DH Gunn TP. Self-efficacy and skill development: influence of task strategies and attributions. J Educ Res. 1986;79(4):238-244.

[64] Speirs JN Lyons MI Johansson BE. Emergency medical service personnel recognize pediatric concussions. Glob Pediatr Health. 2017;4:2333794X17719187. 10.1177/2333794X17719187 PMC5528916 28812053

[65] Tomkinson C Weston E Batt AM. A review of concussion recognition, assessment and management for paramedics. Can Paramed. 2017;40(5):25-27.

[66] Tresolini CP Stritter FT. An analysis of learning experiences contributing to medical students’ self-efficacy in conducting patient education for health promotion. Teach Learn Med. 1994;4:247-254.

[67] van Dinther M Dochy F Segers M . Factors affecting students’ self-efficacy in higher education. Educ Res Rev. 2011;6(2):95-108.

[68] Wallace J Beidler E Covassin T. Assessment and management of sport-related concussion teaching trends in athletic training programs. Athl Train Educ J. 2018;13(2):112-119.

[69] Walrand S Gaulmin R Aubin R Sapin V Coste A Abbot M. Nutritional factors in sport-related concussion. Neurochirurgie. 2021;67(3):255-258. 33582206 10.1016/j.neuchi.2021.02.001

[70] Weber ML Dean JL Hoffman NL , et al. Influences of mental illness, current psychological state, and concussion history on baseline concussion assessment performance. Am J Sports Med. 2018;46(7):1742-1751. 29672135 10.1177/0363546518765145

[71] Xi D McCombe G Agarwal G , et al. Paramedics working in general practice: a scoping review. HRB Open Res. 2021;4:34.

[72] Yorke AM Littleton S Alsalaheen BA. Concussion attitudes and beliefs, knowledge, and clinical practice: survey of physical therapists. Phys Ther. 2016;96(7):1018-1028. 26637654 10.2522/ptj.20140598

