# Supplementary material for: Concussion Assessment and Management Self-efficacy Among Irish Clinicians
Source: Sports Health. 2024 Oct 24;17(4):710–22. doi: 10.1177/19417381241287209 (PMC12188122; doi:10.1177/19417381241287209)
Supplement: sj-pdf-1-sph-10.1177_19417381241287209 – Supplemental material for Concussion Assessment and Management Self-efficacy Among Irish Clinicians [file sj-pdf-1-sph-10.1177_19417381241287209.pdf]

Descriptive information

Q1. What is your age in years? (Dropdown menu provided)

Q2. What is your gender?

- Man
- Woman
- Non-binary
- Prefer not to say
- Other: ___________________________

Q3. Are you a practicing medical or healthcare professional practicing in Ireland?

- Yes
- No

The survey will end for participants that choose 'No'.

Q4. What was the field of your undergraduate study? (Tick all that apply)

- Athletic therapy
- Health Science/Nutrition
- Medicine
- Nursing
- Paramedic studies
- Physiotherapy
- Occupational therapy
- Sports Science/Exercise Physiology
- Other: ____________________

Q5. Was assessment (i.e. diagnosis or recognition) of concussion included in any of your undergraduate study modules?

- Yes
- No

Q6. Was management (i.e. advice, treatment, rehabilitation or referral) of concussion included in any of your undergraduate study modules?

- Yes
- No

Q7. How long ago did you graduate from the undergraduate degree (in years)? (Dropdown menu provided)

Q8. Did you study at a postgraduate level?

- Yes
- No

Participants will be redirected to Q13 if they choose the 'No' option.

Q9. What was the field of your postgraduate study?

- Athletic therapy
- Medicine
- Nursing
- Occupational therapy
- Physiotherapy
- Sports and exercise medicine
- Sports physiotherapy
- Sports rehabilitation
- Other: ______________________

Q10. Was assessment (i.e. diagnosis or recognition) of concussion included in any of your postgraduate study modules?

- Yes
- No

Q11. Was management (i.e. advice, treatment, rehabilitation or referral) of concussion included in any of your postgraduate study modules?

- Yes
- No

Q12. How long ago did you graduate from the postgraduate degree (in years)? (Dropdown menu provided)

Q13. What are your professional qualifications? (Tick all that apply)

- Certified Athletic Therapist
- Chartered Physiotherapist
- Doctor of Medicine
- Occupational Therapist
- Paramedic
- Registered Nurse
- Other: _________________

Q14. Are you currently working as a medical or healthcare professional with sporting populations?

- Yes
- No

Participants will be redirected to Q18 if they choose the 'No' option.

Q15. How long have you been working as a medical or healthcare professional with sporting populations, in years? (Dropdown menu provided)

Q16. What percentage of your patients present with sports injuries? (Dropdown menu provided)

Q17. What sporting populations do you work with? (Tick all that apply)

- Community sport
- Elite sport
- Children
- Adolescents
- Adults

Q18. Do you assess and/or manage concussion as a part of your clinical practice?

- Yes
- No

Q19. How many patients on average do you assess and/or manage for concussion annually? (Dropdown menu provided)

Q20. Have you ever participated in a concussion focused continuing professional development (CPD) event?

- Yes
- No

Participants who choose 'No' will be redirected to the next section.

Participants who choose 'Yes' will answer Q20 before being redirected to the next section.

Q21. How many times have you participated in a concussion focused continuing professional development (CPD) event? (Dropdown menu provided)

<u>Factors impacting confidence</u>

A number of factors that could have a <u>positive impact</u> on the development of confidence are listed below. Please consider how much the following factors facilitated or positively impacted your confidence in concussion assessment and management.

**Factors**	No positive impact at all	Little positive impact	Mild positive impact	Significant positive impact	Very significant positive impact	Not applicable (N/A)
Q41. Practicing the techniques in class						
Q42. Practicing the techniques in clinical placement						
Q43. Practicing the techniques while working independently after graduation						
Q44. Observing a lecturer/placement supervisor performing the techniques						
Q45. Observing a peer/fellow student performing the techniques						
Q46. Being verbally encouraged by a lecturer/placement supervisor, that you can successfully perform the techniques						
Q47. Being verbally encouraged by a peer/fellow student, that you can successfully perform the techniques						
Q48. Being physically calm and collected when practicing the techniques						
Q49. Being emotionally calm and collected then practicing the techniques						
Q50. Receiving feedback on positive aspects of your performance, provided by a lecturer/placement supervisor						
Q51. Receiving feedback on positive aspects of your performance, provided by a peer/fellow student						

Q52. Receiving feedback on negative aspects of your performance, provided by a lecturer/placement supervisor						
Q53. Receiving feedback on negative aspects of your performance, provided by a peer/fellow student						

A number of factors that could have a negative impact on the development of confidence are listed below. Please consider how much the following factors acted as barriers or negatively impacted your confidence in concussion assessment and management.

**Factors**	No negative impact at all	Little negative impact	Mild negative impact	Significant negative impact	Very significant negative impact	Not applicable (N/A)
Q54. Being unable to practice the techniques in class						
Q55. Being unable to practice the techniques in clinical placement						
Q56. Being unable to practice the techniques while working independently after graduation						
Q57. Being unable to observe a lecturer/placement supervisor performing the techniques						
Q58. Being unable to observe a peer/fellow student performing the techniques						
Q59. Receiving no verbal encouragement from a lecturer/placement supervisor, that you can successfully perform the techniques						
Q60. Receiving no verbal encouragement from a peer/fellow student, that you can successfully perform the techniques						
Q61. Being physically agitated (i.e. experiencing increased heart rate, sweating) when practicing the techniques						

Q62. Being emotionally stressed and anxious when practicing the techniques						
Q63. Receiving no feedback on positive aspects of your performance, from a lecturer/placement supervisor						
Q64. Receiving no feedback on positive aspects of your performance, from a peer/fellow student						
Q65. Receiving no feedback on negative aspects of your performance, from a lecturer/placement supervisor						
Q66. Receiving no feedback on negative aspects of your performance, from a peer/fellow student						

Confidence, learning experience and current practices

A number of concussion assessment, reassessment and management techniques are listed in the table below. Please respond to each listed technique according to the following instructions.

Fist column: Rate how certain you are that you can correctly perform each of these techniques and use their findings to guide your clinical decisions in concussion assessment, reassessment and management.

Rate your degree of confidence by recording a number from 0 to 100 using the scale given below:

0	10	20	30	40	50	60	70	80	90	100
Cannot do at all					Moderately can do					Highly certain can do

Please note that you can choose any number from 0 to 100, i.e. 43, 57, 78 etc.

Second column: Rate how often you use the techniques with concussed patients in your clinical practice.

Rate that frequency by recording a number from 0 to 100 using the scale given below:

0	10	20	30	40	50	60	70	80	90	100
Never use the technique					Use the technique with half of concussed athletes					Use the technique with every concussed athlete

Please note that you can choose any number from 0 to 100, i.e. 43, 57, 78 etc.

Third column: Record whether or not the technique was included in the curriculum of your professional education or continuing professional development (CPD) courses.

Y – Yes; N – No; U – Unsure

Assessment/reassessment techniques	Confidence (0-100)	Frequency of use in your clinical practice (0-100)	Included in your professional education curriculum or continuing professional development (CPD) course (Y/N/U)
Assessment of concussion relevant health history (e.g. previous concussions, ADHD, learning difficulties, migraines)			
History and clinical evaluation non-specific to concussion (e.g. cervical ROM, neck strength, myotomes/dermatomes)			
Cervical spine tests (e.g. cervical joint-reposition error test, smooth-pursuit neck torsion test)			
Cranial nerve examination			
Any concussion symptom checklist			
Standard Assessment of Concussion (SAC)			
Sport Concussion Assessment Tool (SCAT 5)			
Child version of Sport Concussion Assessment Tool (Child SCAT5)			
Balance measure (e.g. BESS)			
Gait measure (e.g. timed tandem gait)			
Vestibular/Ocular Motor test (e.g. VOMS)			
King-Devick Test			
Paper/pencil neuropsychological test (i.e The Symbol Digit Modalities Test)			
Computerized neuropsychological test (e.g. ImPACT)			

Reaction time testing not included in computerised neuropsychological testing (e.g. ruler drop test)			
Aerobic exercise tolerance test (e.g. Buffalo Concussion Treadmill Test)			
Mood, anxiety or depression assessment (e.g. Brief Symptom Inventory-18)			
Sleep quality and quantity measure (e.g. Pittsburgh Sleep Quality Index)			
Migraine assessment (e.g. The Migraine Disability Assessment)			

**Management, treatment or rehabilitation technique**	**Confidence (0-100)**	**Frequency of use in your clinical practice (0-100)**	**Included in your professional education curriculum or continuing professional development (CPD) course (Y/N/U)**
Providing advice on cognitive rest			
Providing advice on physical rest			
Providing advice on use of medications			
Providing advice on nutrition			
Providing advice on driving			
Providing advice on return to school/learning activities			
Prescription of aerobic exercise			
Return to play progression (as per consensus statements e.g. graduated stepwise progression)			
Balance training			

Cervical spine rehabilitation			
Treatment of chronic headache			
Vestibular/Ocular Motor rehabilitation			
Referral to a specialist (e.g. optometrist, vestibular specialist, psychologist)			

The survey will end after this section and the following statement will be displayed

Thank you for taking time to complete this survey.
